# New Onset British Accent, Acute Behavioral Changes, and Seizures: A Unique Presentation of NMDAR Encephalitis

**DOI:** 10.1155/2019/2961874

**Published:** 2019-06-18

**Authors:** Mohankumar Kurukumbi, Tulsi S. Shah, Jose A. Castillo, Rahul U. Nayak, Jahnnavi Madiraju

**Affiliations:** ^1^Department of Neurology, Inova Fairfax Hospital, Falls Church VA, USA; ^2^Virginia Commonwealth University School of Medicine, Inova Campus, Falls Church VA, USA

## Abstract

The leading cause of autoimmune encephalitis is N-methyl-D-aspartate receptor (NMDAR) encephalitis. Symptoms can present as prominent behavioral abnormalities prompting inaccurate psychiatric diagnoses. Psychiatric features such as bizarre behavior, agitation, anxiety, delusions, and hallucinations are well noted in the current literature, but a manifestation of foreign accent syndrome has, to our knowledge, never been reported in cases of encephalitis. Once diagnosed, initiation of therapy can result in effective treatment. Here, we present a case of a 32-year-old female with new onset seizures and marked behavioral changes, such as speaking in a foreign accent, who was empirically treated for NMDAR encephalitis due to strong clinical suspicion, showed no improvement with first line therapy with IVIG and IV steroids, and finally had rapid resolution of symptoms with the early initiation of second line therapy of rituximab. In a young female presenting with nonspecific behavioral changes, NMDAR encephalitis should be on the differential and, although CSF antibodies are definitively diagnostic, there should be a low threshold to start empiric therapy and escalate to second line treatment.

## 1. Introduction 

NMDAR encephalitis is one of the most common autoimmune encephalitis syndromes; however symptoms can often be difficult to distinguish from psychiatric etiologies. In addition, it often takes time for definitive diagnostic lab results, which can be detrimental given that research has shown that early intervention can lead to a better prognosis and decreased risk of relapse. Once the treatment has been initiated, the ideal timeline of escalation to second line therapies has not been completely elucidated. Here we present an interesting case of a 32-year-old female with new onset seizure activity and multiple acute behavioral changes, including a rare finding of foreign accent syndrome, found to have NMDAR encephalitis which was resolved with empiric treatment and early escalation to second line therapy.

## 2. Case Presentation 

A 32-year-old female with a history of hyperthyroidism status after radiation resulting in hypothyroidism and no history of seizures presented with an acute onset of behavioral changes and witnessed seizure activity. Family history is remarkable for thyroid disease in multiple relatives, but negative for seizure or psychiatric disorders. Behavioral changes included uncontrolled laughter, screaming, signs of agitation, spitting on the floor, complete lack of appetite, and speaking in a British accent. A further history revealed that the patient is of Caucasian descent, was born in Germany, moved to the United States when she was a baby, and has no ties to Britain.

**Figure 1:**
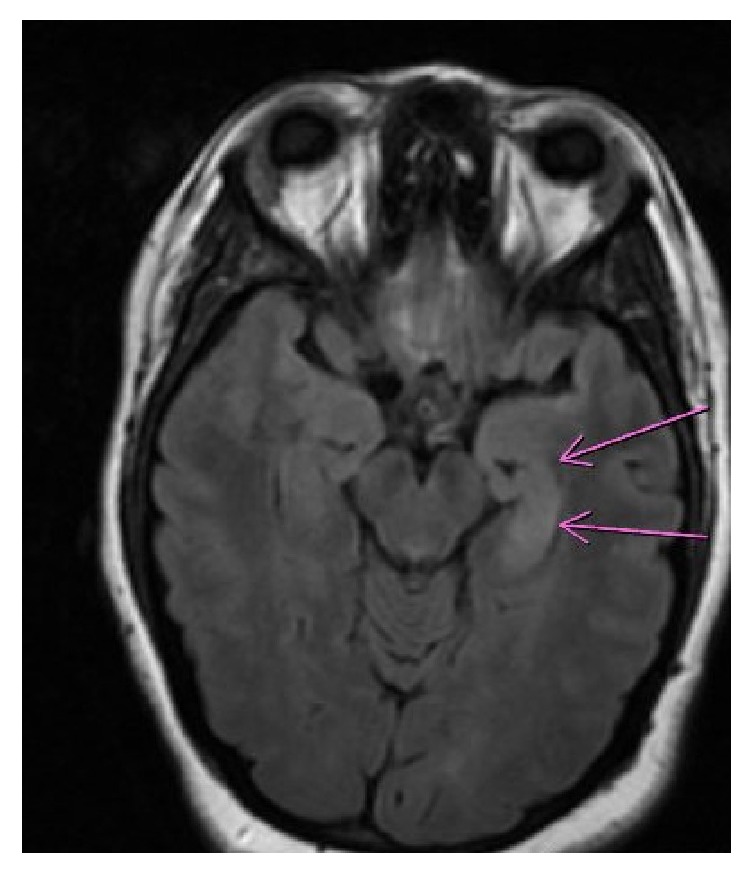
Asymmetric increased flair signal over the left hippocampus.

She had two seizures both involving tongue biting and postictal confusion with combative behavior. Her workups for seizures, including but not limited to head CT, urine drug screen, and electrolyte levels, were all within normal limits. She, as well as her family, refused MRI and subsequently was discharged on levetiracetam for new onset seizures. On the fourth day of illness (DOI), the patient was admitted to a local community hospital with continued behavioral changes, where an MRI and lumbar puncture (LP) were found to be unremarkable, with a WBC count of 1 cell per mm^3^. Other CSF parameters include a RBC count of 10 cells per mm^3^, glucose of 53 mg per dL, and protein of 26 mg per dL. She was subsequently transferred to our hospital care on the fourteenth day of illness because of persisting and worsening behavioral changes. Upon revisiting the initially unremarkable impression of the MRI, abnormal T2 flair hyperintensity in the mesial temporal lobes was noted, with left lobe hyperintensity greater than that of the right lobe (Figure 1). These features raised suspicion for limbic encephalitis.

**Figure 2:**
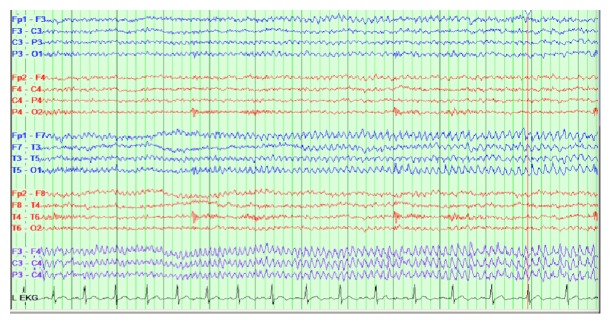
Electroclinical seizure: rhythmic theta discharges originating from left frontocentral/ frontotemporal region.

**Figure 3:**
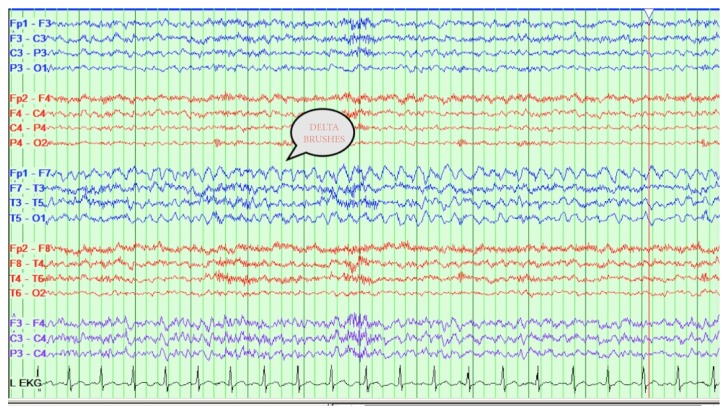
Electroclinical seizure: rhythmic delta activity originating from left frontotemporal region.

Titers for serum anti-NMDAR and paraneoplastic antibody panel were sent on clinical suspicion. Video EEG (Figure 2) showed frequent focal onset electrographic seizures from the left frontocentral and left frontotemporal region. Some of these electroclinical seizures showed delta brushes (Figure 3). With strong clinical suspicion for an anti-NMDAR or paraneoplastic antibody related encephalitis, before even receiving antibody titer results, the patient was started on five-day IV steroids on the fourteenth DOI, and IVIG course started on the fifteenth DOI.

At this point, multiple differential diagnoses were being considered including autoimmune encephalitides. During hospital admission, the patient continued to remain afebrile. Repeat LP showed lymphocytic pleocytosis, with a quantitative value of 54 cells per mm^3^ with 98% lymphocytes, after which she was started on empiric acyclovir until HSV PCR was later confirmed to be negative. All other CSF findings were insignificant, with a normal protein level of 16.8 mg per dL, no RBCs, and an elevated glucose level of 95 mg per dL. She continued to have seizures requiring Lacosamide followed by an addition of Lamotrigine. On the fifth day of IVIG course and twentieth DOI, NMDAR antibody was found to be positive in the serum. CT of the chest, abdomen, and pelvis showed no evidence for neoplasms and a transvaginal ultrasound was negative for ovarian teratoma. CSF was positive for NMDAR antibody with a titer of 1:64. After completion of IV steroids and IVIG, there was no significant clinical improvement. She was started on rituximab on DOI 27 for a total of 4 weeks given weekly, with subsequent clinical improvement in addition to no clinical seizures on EEG and resolution of her new onset British accent.

The patient continued to have subclinical focal seizures, catatonia, and orofacial dyskinesias with subsequent gradual improvement in her behavior at discharge. When seen for follow-up as an outpatient, she still was found to have some residual memory and cognitive processing deficits. The patient continues to follow up in the hospital for rituximab infusions which is controlling her anti-NMDAR encephalitis and she has now returned back to baseline health.

## 3. Discussion 

According to epidemiologic studies, NMDAR encephalitis is not only the best characterized of autoimmune encephalitis syndromes, but also the most common. The California Encephalitis Project, established in 1998 to better understand the epidemiology of encephalitis and its etiologies, found that, of the 761 cases of uncertain etiologies of encephalitis, the leading cause was by far anti-NMDAR encephalitis, present in 32 of 79 identified etiologies. Anti-NMDAR encephalitis was identified more than 4 times more frequently than HSV-1, West Nile virus, or Varicella Zoster virus [1].

Interestingly, anti-NMDAR receptor encephalitis was initially thought to be solely a paraneoplastic disorder associated with young females with immature ovarian teratomas [1]. However, further studies revealed that approximately 80% of patients with anti-NMDAR encephalitis are women, and, in women above the age of 18, only about 50% have an ovarian teratoma. Other studies also confirmed that it can occur in both males and females, children and adults, and is not in fact always associated with a neoplastic cause [2], as in our case. In our patient, CT abdomen/pelvis and transvaginal ultrasound were both negative, likely excluding the presence of a teratoma. Though retrospectively, we recognize that a pelvic MRI is most sensitive for teratoma screening; this imaging modality was not done in our patient.

In NMDAR encephalitis, it has been shown that the NMDAR antibodies cause increased internalization of the receptor leading to effective loss of NMDAR signaling via disruption of the GluN1/N2b interaction with Ephrin-B2. [1, 3]

Many patients initially present with headache, fever, or a viral-like condition, which in a few days usually progress to include prominent psychiatric features such as bizarre behavior, agitation, anxiety, delusions, and hallucinations. In addition, other characteristic clinical symptoms of anti-NMDAR encephalitis include seizures, stupor, autonomic instability, dyskinesias, memory deficits, and language dysfunction [4]. A manifestation of foreign accent syndrome, as seen in our patient, has to our investigation never been reported.

Interestingly, Dalmau et al. reported in a case study of 100 participants that 77 were initially evaluated by psychiatry [4]. This can make properly identifying anti-NMDAR encephalitis symptoms difficult. NMDAR encephalitis is a differential that must always be kept in mind when a young female acutely presents with psychiatric symptoms.

From all of the possible manifestations of anti-NMDAR encephalitis, our patient presented with seizures, bizarre behavior, agitation, and a change in language function. Interestingly our patient was using a British accent upon presentation.

This phenomenon, known as foreign accent syndrome (FAS), is well appreciated with functional or structural neurologic disorders like conversion disorders and strokes, but not as a manifestation of encephalitis.

FAS is a rare disorder where the affected person speaks in an accent that the listener perceives as foreign. Although most cases have left hemisphere lesions, some may be functional. It was first described by French neurologist Pierre Marie in 1907 with a patient who was recovering from a left hemisphere subcortical stroke. Since then there have been over 60 reported cases with etiology consistent with stroke or traumatic brain injury and a few associated with underlying psychological disorder. Interestingly, neither was the case in our patient as the diagnosis was that of an inflammatory process and not a structural or psychological one.

Currently the cause behind this syndrome is unknown. It has been proposed that it results from lesions of the dominant language cortex (usually the left hemisphere). Our patient had only mesial temporal lobe involvement found via MRI.

There are many types of speech disorders, including stuttering disfluencies, speech substitutions, and defects in the rhythm and sound of speech, which could present in the form of a foreign accent [5, 6]. The current literature shows reports of various foreign accents including Welsh and French, and, now in our case, a patient with a British accent.

This syndrome is diagnosed clinically. MRI findings can be used in support of a structural cause for the syndrome, like a stroke or traumatic brain injury [5].

Currently no proven treatment options exist for this phenomenon, besides attempting to correct the underlying structural or functional pathology and pursuing supportive measures like speech therapy or insight-oriented counseling [6]. Most reported cases endorse resolution of the foreign accent with time but some also mention continued periods of foreign accent speech. Though our patient initially presented with a British accent, these findings completely resolved by the end of her clinical course.

MRI is helpful in the clinical setting but findings are neither unique nor diagnostic for this disorder. A MRI of the brain is abnormal in only 30-50% of patients with NMDAR encephalitis [3]. Whether or not an MRI is more likely to indicate a pathologic process if done at a certain time of the disease course has not been definitively studied. However, one study found that, with initially normal MRI findings, further findings tend to remain normal or show minimal changes, despite the severity and duration of symptoms [2].

If it is indicative of an underlying pathology, a T2-weighted fluid-attenuated inversion recovery (flair) brain MRI frequently shows hyperintense signals in the grey and/or white matter, and commonly present in the medial temporal lobes [4].

A continuous video EEG mainly excludes a nonconvulsive seizure. Anti-NMDAR encephalitis shows some common nonspecific EEG patterns which include epileptiform activity, focal and/or generalized slowing, and periodic lateralized epileptiform discharges [7]. A unique finding in approximately one-third of people affected by anti-NMDAR encephalitis is a pattern in the EEG tracing called extreme delta brush, that is now becoming recognized as characteristic for anti-NMDAR encephalitis [8]. Our patient had EEG findings indicative of anti-NMDAR encephalitis, including focal slowing and delta brush, as seen in one of the figures.

The sensitivity of anti-NMDAR antibody testing was found to be higher in the CSF when compared to serum [9]. The diagnosis of anti-NMDAR encephalitis is therefore confirmed by the presence of antibodies, specifically immunoglobulin G (IgG), to the NMDAR's GluN1 subunit in the CSF. 

The mainstay treatment option for anti-NMDA receptor encephalitis is immunosuppression [10]. Most patients have a favorable prognosis when treatment is started early [11]. Particularly, improvement of the prognosis probability was higher with administration of immunotherapy at <40 days of illness when compared to >40 days; however, comparison for earlier dates has yet to be studied [11]. Evidently in our case, treatment was started on the fourteenth DOI.

One study found that patients with delays in diagnosis and treatment are more likely to have persistent impairments, such as deficits in executive function and memory, when compared to those with early intervention [12]. Our patient on follow-up as an outpatient initially had some residual deficits but showed gradual improvement in her behavior and with continuous follow-up with rituximab infusions showed complete resolution.

The literature for treatment is in support of an escalation approach with promise being evident when the first line agents being used are steroids and IVIG or plasma exchange [10].

The regimen most frequently used is a combination of steroids and IVIG. The literature regarding whether the efficacy of IVIG or plasma exchange is equivalent to treating anti-NMDAR encephalitis has yet to be further explored. If there is no evidence of improvement with first line agents, second line therapies should be administered including rituximab, cyclophosphamide, or a combination of both. One series confirmed this by showing that 75-80 percent of patients showed improvement by 24 months when this was the case [10].

In this case the patient's symptoms were refractory to IVIG and steroids. Second line therapy of rituximab was started and showed significant clinical improvement, and therefore the patient was continued on rituxan infusions.

Management and treatment of anti-NMDAR encephalitis still hold a lot of uncertainties, as timeline, duration, combination, and efficacy of treatment options are still unclear. However, it is clear that management should not be limited to first line agents and if the patient is not showing improvement of symptoms, second line therapies should always be taken into consideration and can completely change the clinical picture.

## 4. Conclusion 

This case is reported due to the unique presentation of anti-NMDAR encephalitis, which included a never before seen symptom of FAS that resolved with correction of the underlying pathology. FAS may apparently also be of autoimmune or inflammatory etiology.

For any patient who presents with psychiatric manifestations with no underlying psychiatric history and seizures, it is vital to consider anti-NMDAR encephalitis as well as CSF studies in the workup. Additionally, it is important to note that management should not be limited to IVIG and steroid therapy; rituximab infusions should be a secondary treatment if deemed necessary.

## Abbreviations

CT: Computed tomography
EEG: Electroencephalogram
MRI: Magnetic Resonance Imaging
LP: Lumbar puncture
NMDA: N-methyl-D-aspartate
NMDAR: N-methyl-D-aspartate receptor
IV: Intravenous
IVIG: Intravenous immunoglobulin
HSV-1: Herpes Simplex Virus-1
FLAIR: Fluid-attenuated inversion recovery
VGKC: Voltage gated potassium channel
WBC: White blood cell
AED: Antiepileptic drug
CSF: Cerebrospinal fluid
IgG: Immunoglobulin G
DOI: Day of illness.
